# Arsenic exposure and risk of skin cancer (melanoma and non-melanoma): A systematic review and meta-analysis^☆^

**DOI:** 10.1016/j.abd.2025.501161

**Published:** 2025-07-17

**Authors:** Lijiao He, Meiying Wei, Qikui Yang, Yun Huang, Zuyuan Wei

**Affiliations:** Department of Dermatology, Wenshan Zhuang and Miao Autonomous Prefecture Hospital of Traditional Chinese Medicine, Wenshan, China

**Keywords:** Arsenic, Basal cell carcinoma, Melanoma, Meta-analysis, Squamous cell carcinoma, Systematic review

## Abstract

**Background:**

Arsenic, recognized as a potentially lethal substance and a carcinogen, has been associated with an increased risk of skin cancer; however, the findings have been inconsistent. The aim of this study was to assess the impact of arsenic exposure on skin cancer risk (including melanoma and non-melanoma) through a meta-analysis of the available data.

**Objectives:**

To assess the risk of skin cancer from arsenic exposure.

**Methods:**

Searches were performed in databases such as PubMed, Web of Science, Embase, and CNKI (as of June 10, 2024). The pooled odds ratio (OR) and its 95% Confidence Interval (95% CI) were calculated using a random effects model. Subgroup analyses were performed considering sample size, study centers, U.S. regions, arsenic exposure routes, and measurement methods.

**Results:**

A total of 12 papers were included, comprising 48,003 participants. The findings indicated an association between arsenic exposure and the risk of skin cancer ([OR = 1.51], 95% CI 1.26–1.80). Specifically, the OR was 1.52 (95% CI 1.06–2.17) for melanoma, 1.64 (95% CI 1.16–2.32) for squamous cell carcinoma, and 1.36 (95% CI 1.04–1.77) for basal cell carcinoma. Subgroup analyses also revealed an association between arsenic exposure and skin cancer in the United States (OR = 1.52, 95% CI 1.25–1.87). Both ingestion and inhalation pathways of arsenic exposure showed a trend toward an increased risk of skin cancer.

**Study limitations:**

An important limitation of this study is a degree of heterogeneity, and another is due to the limited number of research papers available.

**Conclusion:**

This meta-analysis indicates that arsenic exposure may be associated with an elevated risk of skin cancer. Additional prospective research is necessary to verify the association between arsenic exposure and the incidence of skin cancer, encompassing both cutaneous malignant melanoma and non-melanoma skin cancer.

## Introduction

Skin cancers can be categorized into two main groups: cutaneous malignant melanoma and non-melanoma skin cancer (NMSC). NMSC, predominantly formed of squamous cell carcinoma (SCC) and basal cell carcinoma (BCC), is the most commonly identified cancer, making up approximately one-third of all malignant tumors diagnosed globally each year.^1^, ^2^ BCC represents almost 80% of all NMSC cases detected each year,^3^ with SCC making up the remaining 20%.^4^, ^5^ Melanoma, an aggressive type of skin cancer arising from melanocytes, accounts for under 5% of all skin cancer cases but poses a substantial threat. If left untreated, melanoma, responsible for 75% of all deaths related to skin cancer, has the ability to metastasize to different areas of the body.^6^, ^7^ Collectively, melanoma and NMSC impose a substantial economic and health burden, which is anticipated to keep increasing in the future.

Arsenic has been used as a medicine (Fowler's solution) for the treatment of syphilis, malaria and psoriasis for the past two centuries.^8^ However, after 30-years of treatment, patients developed multiple skin cancers.^9^ Subsequent research has progressively demonstrated that long-term chronic exposure to arsenic can result in a range of cancers, including skin, lung,^10^, ^11^ and, to a lesser extent, liver, kidney, and bladder cancers.^12^, ^13^, ^14^ Consequently, many arsenic-containing drugs have been restricted or phased out entirely. The International Agency for Research on Cancer (IARC) classifies arsenic as a Group I human carcinogen.^15^ Nonetheless, Arsenic is commonly found in soils, sediments, and groundwater, either occurring naturally or as a result of human activities such as food preparation, industrial processes, mining and pesticide use. Humans can be exposed to arsenic through various pathways.^16^ According to recommendations from the World Health Organization (WHO), the level of arsenic in drinking water should not exceed 10 μg/L.^17^ However, this guideline is often increased to 50 μg/L in numerous developing nations.^18^ Globally, more than 100 million individuals are at risk of consuming arsenic levels over 50 μg/L in their drinking water.^19^ Average dietary exposure to inorganic Arsenic (iAs) ranges from 0.1 to 3.0 μg/kg per day in Europe, Asia, and the United States.^20^ The National Research Council (NRC) Risk Assessment from 2001 suggests that even exposure to lower concentrations of arsenic poses a relatively high risk of cancer.^21^

Extensive research has been conducted on the association between exposure to arsenic and NMSC.^22^, ^23^, ^24^, ^25^, ^26^, ^27^, ^28^ Multiple epidemiological investigations have established a association between exposure to arsenic and a heightened likelihood of developing NMSC in different locations such as Taiwan, Mexico, Bangladesh, and Chile.^29^, ^30^, ^31^ Research in Taiwan^31^, ^32^ revealed a significant dose-response relationship for this association, while other studies did not observe such an effect.^23^, ^25^, ^26^, ^28^ Conversely, research on the potential association between melanoma and exposure to arsenic has yielded divergent findings.^22^, ^33^, ^34^, ^35^, ^36^, ^37^, ^38^ While some studies indicate a possible association between arsenic exposure and a heightened likelihood of developing skin cancer, the definitive nature of this link is still debated. To investigate this potential association more thoroughly, a meta-analysis was carried out to systematically review existing data concerning arsenic exposure and its association to skin cancer, encompassing both melanoma and non-melanoma cases.

## Methods

### Protocol

The meta-analysis was registered with PROSPERO (CRD42024556618) and adhered to the PRISMA guidelines for thorough reporting.^39^ Supplementary Appendix 1 contains the PRISMA checklist. Approval from the institutional review board was not necessary for this study, as it consisted of a systematic review and meta-analysis of existing literature.

### Literature search strategy

Two researchers, Li Jiao He and Mei Ying Wei, individually searched PubMed, Embase, Web of Science, Cochrane Library, and CNKI (as of June 10, 2024). Key terms such as arsenic, basal cell carcinoma, squamous cell carcinoma, and melanoma were employed. Search expansion was conducted using mesh terms in PubMed, such as Malignant Melanoma, Arsenic-75, Epidermoid Carcinoma, and Rodent Ulcer. No limitations were placed on language or time in the search strategy. Each author conducted an independent review of all titles and chose relevant abstracts, while duplicates and unrelated articles were excluded. Final decisions on study inclusion or exclusion were made by consensus. The analysis included studies that examined the association between exposure to arsenic and skin cancer, encompassing both melanoma and non-melanoma (Specific search strategies are supplemented in Supplementary Appendix 2).

### Inclusion and exclusion criteria

The criteria for including studies were defined as: 1) Case-control or prospective cohort research designs; 2) Studies involving populations with melanoma, BCC, SCC, or corresponding control groups; 3) Documentation of arsenic exposure history; 4) Availability of 95% Confidence Intervals (95% CIs) or sufficient data to calculate Odds Ratios (ORs) or Relative Risks (RRs) for the study outcomes.

The exclusion criteria for this study included: 1) Insufficient data or results; 2) Non-comparative studies, in vitro experiments, animal experiments, case reports, path mechanisms, conference abstracts, letters, reviews, and expert opinions; and 3) Individuals who were not diagnosed with skin cancer.

### Data extraction

Two researchers independently reviewed the literature based on inclusion and exclusion criteria and collected data with a standardized form for information extraction. The data retrieved was verified by both researchers, and inconsistencies were addressed by a third-party specialist. Each study provided the following details: 1) Study details, including study design, country, the follow-up duration, study population (number of cases and controls or cohort members), and demographic characteristics. 2) Type of arsenic exposure (e.g., water, dust, biological samples, questionnaires). 3) Definitions of outcomes, such as melanoma, BCC, and SCC (including histological diagnosis and ICD codes). 4) Reported effect measures, including odds ratios, relative risks, and corresponding 95% Confidence Intervals for exposed and unexposed groups.

### Quality assessment and risk of bias

The quality of these studies was thoroughly evaluated by two reviewers utilizing the Newcastle-Ottawa Scale (NOS).^40^ This assessment focused on three primary criteria: study selection, comparability of exposures, and evaluation of outcomes. The revised NOS utilizes a 9-star rating system: research is evaluated with 1–3 stars for poor quality, 4–6 stars for medium quality, and 7–9 stars for excellent quality. Two independent reviewers utilized the Cochrane Non-Randomized Study Intervention (ROBINS-I) tool to evaluate the risk of bias in the selected studies.^41^ During the assessment process, the authors classified the risk of bias into five levels: low, moderate, serious, critical, or insufficient information, based on seven domains: 1) Bias due to Cofounding; 2) Bias in deviation from intended outcomes; 3) Bias in classification of interventions; 4) Bias due to deviation from intended intervention; 5) Bias due to missing data; 6) Bias in measurement of outcomes; 7) Bias in selection of the reported results. If at least one domain was rated as high or moderate, the overall risk of bias was classified as high or moderate; otherwise, it was classified as low. Any disagreements between the two investigators were resolved unanimously through consensus.

### Statistical analysis

Stata SE 15.0 software was utilized to conduct statistical analysis for the computation of Odds Ratio (OR) and the corresponding 95% Confidence Intervals (95% CI) pertaining to binary variables. The choice between fixed-effects and random-effects models was based on the I² index and Cochran *Q* test p-values.^42^ Heterogeneity was categorized as low (I² < 25%), moderate (25 %–75 %), or high (I² > 75%).^43^ Significant heterogeneity was determined by a Cochran *Q* test p-value < 0.05. A subgroup analysis was carried out to examine the origins of variability, and a sensitivity analysis was done by removing individual studies to gauge the stability of the outcomes. The assessment of publication bias was done by visually inspecting the asymmetry of the funnel plot and using the Begg test.^44^ All statistical tests were conducted as two-sided tests, with a significance level set at p < 0.05.

## Results

### Results of the literature search

**Fig. 1:**
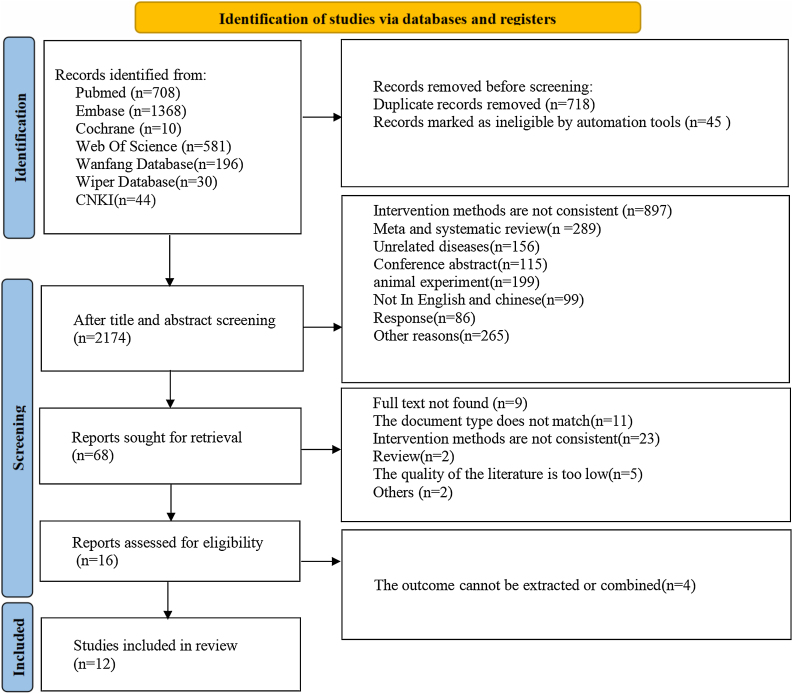
Flowchart of study retrieval for this meta-analysis.

Initially, a total of 2937 articles were retrieved through the literature search. Following the exclusion of 718 duplicate entries, an additional 2106 articles were removed according to the predefined criteria. Initially, 68 studies underwent title and abstract review. Upon full-text assessment, 56 studies did not meet the inclusion criteria and were subsequently excluded. After thorough examination, 12 research studies met the established criteria and were included in the meta-analysis. The specifics of the screening process for literature and its results are elucidated in Fig. 1.

### Basic characteristics of the included studies

The analysis encompassed 12 studies, consisting of 11 case-control studies and 1 cohort study. Detailed demographic information of the population, such as sex ratio and country of origin, is presented in Table 1. The definitions of arsenic exposure differed across studies, with some emphasizing exposure through drinking water and others highlighting chemical contamination or indirect indicators like toenail and urine arsenic concentrations. These definitions exhibited minor discrepancies among the studies.

**Table 1:** Clinical and demographic characteristics of the included studies in the meta-analysis.

First author	Year	Research type	Author states	Sample (n)		Age (year)		Sex (Male/ Female)		Definitions of arsenic exposure	Follow-up (years)	Skin cancer type
				Skin cancer	Non-skin cancer	Skin cancer	Non-skin cancer	Skin cancer	Non-skin cancer			
Bedaiwi ^33^	2022	Case-control study	USA	Melanoma (87)	Non-skin cancer (12615)	64.6	45.4	M: 51.7% / F: 44.7%	M: 50.0% / F: 50.0%	Urine As concentration (>50ug/L)	NA	Melanoma
Langston ^34^	2022	Case-control study	USA	Melanoma (1096)	Non-skin cancer (1033)	20‒39: 13% 40‒49: 14% 50‒59: 21% 60‒69: 23% 70‒79: 18% 80+: 11%	20‒39: 17% 40‒49: 15% 50‒59: 25% 60‒69: 26% 70‒79: 13% 80+: 4%	M: 52% / F: 48%	M: 45% / F: 55%	Drinking water As (**>**10ug/L)	NA	Melanoma
Beane Freeman ^35^	2004	Case-control study	USA	Melanoma (326)	Non-skin cancer (329)	40‒49: 26% 50‒59: 22.8% 60‒69: 23.1% 70‒79: 19.6% 80‒89: 8.4%	40‒49: 15.8% 50‒59: 29.8% 60‒69: 28.4% 70‒79: 18.2% 80‒89: 7.8%	M: 55.7% / F: 44.3%	M: 64.3% / F: 35.7%	Toenails As concentration **(>**0.084 ug/g)	NA	Melanoma
Collatuzzo ^36^	2023	Case-control study	Italy	Melanoma (295)	Non-skin cancer (293)	<35: 14.8% 35‒49: 27.6% 50‒64: 3.2% ≥65: 24.3%	<35: 20% 35‒49:25.2% 50‒64: 31.2% ≥65: 23.6%	M: 47% / F: 53%	M: 47.2% / F: 52.8%	Occupational exposure to As (undefined)	NA	Melanoma
Dennis ^37^	2010	Cohort study	USA	Melanoma (150)	Non-skin cancer (24554)	57	48	NA	NA	Arsenical pesticide exposure	10.3 years	Melanoma
Kennedy-1 ^22^	2005	Case-control study	Netherlands	Melanoma (47)	Non-skin cancer (164)	NA	NA	NA	NA	Occupational exposure to As(undefined)	NA	Melanoma
Kennedy-2 ^22^	2005	Case-control study	Netherlands	Basal cell carcinoma (249)	Non-skin cancer (164)	NA	NA	NA	NA	Occupational exposure to As (undefined)	NA	Basal cell carcinoma
Kennedy-3 ^22^	2005	Case-control study	Netherlands	Squamous cell carcinoma (103)	Non-skin cancer (164)	NA	NA	NA	NA	Occupational exposure to As (undefined)	NA	Squamous cell carcinoma
Surdu-1 ^23^	2013	Case-control study	USA	Basal cell carcinoma (500)	Non-skin cancer (515)	67	61	M: 44.8% / F: 55.2%	M: 51.6% / F: 48.4%	Occupational exposure to As (dust fumes)	NA	Basal cell carcinoma
Surdu-2 ^23^	2013	Case-control study	USA	Squamous cell carcinoma (70)	Non-skin cancer (515)	71.5	61	M: 54.3% / F: 45.7%	M: 51.6% / F: 48.4%	Occupational exposure to As (dust fumes)	NA	Squamous cell carcinoma
Mitropoulos ^24^	2004	Case-control study	USA	Squamous cell carcinoma (404)	Non-skin cancer (395)	NA	NA	NA	NA	Occupational exposure to As (undefined)	NA	Squamous cell carcinoma
Sánchez ^25^	2013	Case-control study	Colombia	Squamous cell carcinoma (166)	Non-skin cancer (166)	70.8	71.8	M: 31% / F: 69%	M:31% / F: 69%	Occupational exposure to As (carpentry trade wood, gunpowder, metal industries)	NA	Squamous cell carcinoma
Suárez-1 ^26^	2007	Case-control study	France	Basal cell carcinoma (1333)	Non-skin cancer (1507)	60.5	58.2	M: 63% / F: 37%	M: 62% / F: 38%	Occupational exposure to As (Agricultural works)	NA	Basal cell carcinoma
Suárez-2 ^26^	2007	Case-control study	France	Squamous cell carcinoma (183)	Non-skin cancer (1507)	60.5	58.2	M: 63% / F: 37%	M: 62% / F: 38%	Occupational exposure to As (Agricultural works)	NA	Squamous cell carcinoma
Karagas-1 ^27^	2001	Case-control study	USA	Basal cell carcinoma (587)	Non-skin cancer (524)	<40: 7.2% 40‒49:17% 50‒59:21% 60‒69:34.9% ≥70:19.9%	<40: 5.3% 40‒49:12.6% 50‒59:19.7% 60-69:39.3% ≥70:23.1%	M: 57.6% / F: 42.2%	M: 60.1% / F: 39.9%	Toenails As concentration (0.345–0.81 ug/g)	NA	Basal cell carcinoma
Karagas-2 ^27^	2001	Case-control study	USA	Squamous cell carcinoma (284)	Non-skin cancer (524)	<40: 1.1% 40‒49:6 % 50‒59: 16.6% 60‒69: 40.9% ≥70: 35.6%	<40:5.3% 40‒49: 12.6% 50‒59: 19.7% 60‒69: 39.3% ≥70: 23.1%	M: 64.1% / F: 35.9%	M: 60.1% / F: 39.9%	Toenails As concentration (0.345–0.81 ug/g)	NA	Squamous cell carcinoma
Gilbert-Diamond ^28^	2013	Case-control study	USA	Squamous cell carcinoma (470)	Non-skin cancer (447)	<50:4% 50‒59: 19.4% 60‒69: 45.3% ≥70: 31.3%	<50: 7.6% 50‒59: 18.6% 60‒69: 45.4% ≥70: 28.4%	M: 60.4% / F: 39.6%	M: 57.7% / F: 42.3%	Urine As concentration >5.31 ug/L)	NA	Squamous cell carcinoma

M, Male; F, Female; USA, United States of America; non-skin cancer; healthy controls.

### Quality assessment and risk of bias

The Newcastle-Ottawa Scale (NOS) was utilized to assess the quality of the cohort studies included, with the results presented in Table 2. In summary, five articles were rated as high quality, each receiving a score of 7-stars. Among these, two studies focused on the analysis of arsenic levels in urine, two others measured arsenic levels in toenails, and only one investigated arsenic levels in drinking water. These analyses accounted for variables such as age, sex, sun exposure history, and skin type to mitigate potential residual confounding effects. Additionally, a total of seven studies examined the association between occupational arsenic exposure and skin cancer, with five rated as moderate quality (6-stars). These studies employed a semi-quantitative three-stage scale (low, medium, and high) to evaluate occupational arsenic exposure based on intensity, frequency, and probability. Furthermore, the findings adjusted for key confounding factors, including age, sex, skin type, history of solar radiation exposure, family history of cancer, smoking history, and education level. The remaining two studies were rated as relatively low quality (5-stars) due to their reliance solely on data gathered from questionnaires administered by specially trained staff.

**Table 2:** NOS Quality Evaluation Form. The assessment system rates the selection of participants, group comparability, and results evaluation using a 9-star scale. Research is considered poor quality if it is rated 1‒3 stars, moderate quality if it is rated 4‒6 stars, and high quality if it is rated 7‒9 stars.

Study	Selection				Comparability	Outcomes			Total
	1	2	3	4		1	2	3	
Ahmed Bedaiwi	★	★	★		★	★	★	★	7
Marvin E. Langston	★	★	★		★	★	★	★	7
Laura E. Beane Freeman	★	★	★		★	★	★	★	7
Giulia Collatuzzo	★	★	★		★		★	★	6
Leslie K. Dennis	★	★	★		★		★		5
Cornelis Kennedy	★	★	★		★		★	★	6
Simona Surdu	★	★	★		★		★	★	6
Panagiotis Mitropoulos	★	★	★		★		★	★	6
Guillermo Sánchez	★	★	★		★		★		5
Berta Suárez	★	★	★		★		★	★	6
Margaret R. Karagas	★	★	★		★	★	★	★	7
Diane Gilbert-Diamond	★	★	★		★	★	★	★	7

Most studies present a low to moderate risk of bias, mainly due to confounding bias (age, sex, history of solar radiation, skin type), selection bias (high exposure areas), and intervention bias (misclassification of exposure), even though adjustments are made, this concern is particularly salient in observational studies. Two other studies were at serious risk of bias due to bias in the selection of the reported result (see Table S1 in Supplementary Appendix 3).

## Meta-analysis results

### Preliminary analyses

**Fig. 2:**
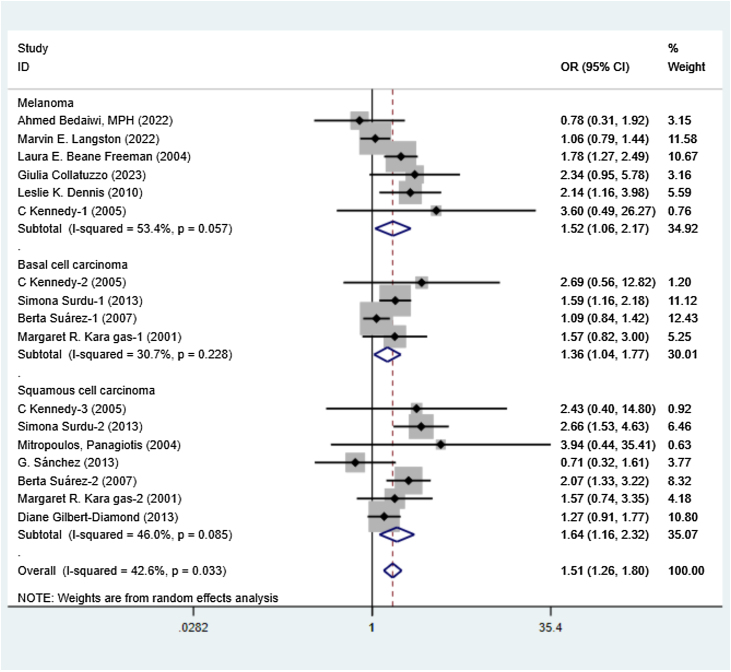
Meta-analysis summarizing the data from various studies that investigate the association between arsenic exposure and the incidence of skin cancer. The weights come from the random-effects model. OR, Odds Ratio; CI, Confidence Interval.

Fig. 2 illustrates the association between exposure to arsenic and the various types of skin cancer, including melanoma, BCC, and SCC, through a forest plot. The analysis was based on 12 studies involving 48,003 participants, with an overall OR = 1.51, 95% CI (1.26–1.80). A meta-analysis was conducted using a random effect model (I² = 42.6%). Specifically, arsenic exposure was associated with melanoma (OR = 1.52, 95% CI 1.06–2.17), SCC (OR = 1.64, 95% CI 1.16–2.32), and BCC (OR = 1.36, 95% CI 1.04–1.77). These results collectively imply that arsenic exposure heightens the risk of these skin cancers. To explore potential sources of variation, additional sensitivity analyses were conducted in light of the observed heterogeneity.

### Sensitivity analyses

**Fig. 3:**
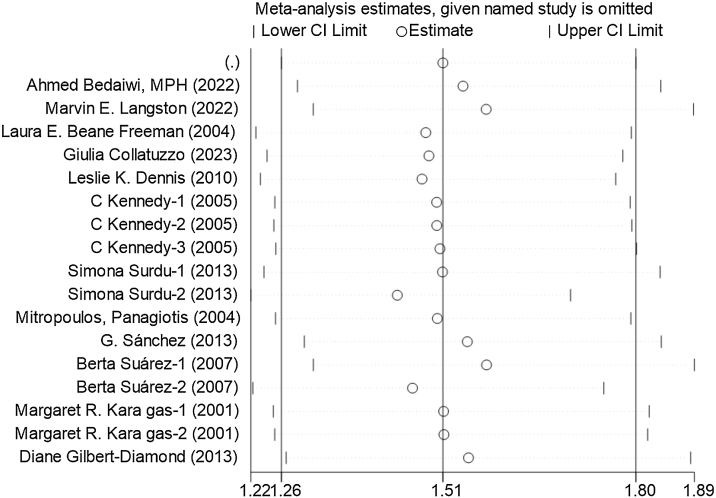
Plot of single-study sensitivity analyses for all studies. Sources of heterogeneity were analyzed using a random-effects model. CI, Confidence Intervals.

A sensitivity analysis was conducted on the studies that were included, as illustrated in Fig. 3. Every individual study was methodically removed from the meta-analysis to assess its influence on the overall risk estimation. The results revealed that the remaining studies consistently converged around an overall OR of 1.51. No significant changes in outcomes driven by individual studies were noted throughout this process. The heterogeneity observed could be attributed to variations in arsenic exposure definitions among the included articles, as well as differences in exposure assessment methods such as water, dust, urine levels, and self-reported exposures. Some studies focused primarily on arsenic in drinking water, while others addressed occupational exposure, chemical contamination, or utilized indirect exposure indicators. Despite these discrepancies, the overall heterogeneity remained moderate, and the meta-analysis model utilized was considered robust and reliable.

### Subgroup analyses

A comprehensive subgroup analysis was conducted across the included studies, considering factors such as sample size, study centers, US regions, arsenic exposure routes, and measurement methods. The analysis revealed a total of 43,469 participants from the U.S. regions, 44,703 participants involved in the measurement methods, and 48,003 participants considered in the analyses of sample size, research centers, and arsenic exposure pathways. The results are shown in Table 3. No significant sources of heterogeneity were identified in these analyses. Subgroup analysis further revealed a significant increase in the risk of skin cancer (both melanoma and non-melanoma) in the US due to arsenic exposure, with a pooled OR of 1.48 (95% CI 1.20–1.83). Whether through food intake (OR = 1.33, 95% CI 1.07–1.64) or inhalation (OR = 1.72, 95% CI 1.23–2.27), there was a consistent trend of increased risk of skin cancer associated with arsenic exposure. In the analysis of the arsenic measurement method (biomarker OR = 1.33, 95% CI 1.07–1.64, occupational assessment semi-definable scale OR = 1.80, 95% CI 1.34–2.42), the results are not biased.

**Table 3:** Subgroup analysis. Sample size, study center, US region, measurement methods, and subgroup analyses of arsenic exposure pathways; weights are from random-effects models.

Subgroup	Study	OR (95% CI)	p-value	I²
**USA**				
Melanoma	3	1.35 (1.10, 1.65)	0.004	65.1
Basal cell carcinoma	2	1.58 (1.19, 2.11)	0.002	0
Squamous cell carcinoma	4	1.54 (1.18, 2.01)	p=0.001	47.8
**Sample size**				
< 100	3	1.64 (1.15, 2.23)	0.006	64.6
> 100	9	1.46 (1.23, 1.72)	p<0.001	35.8
**Exposure route**				
Cconsume	5	1.33 (1.07, 1.64)	0.233	26.9
Inhalation	7	1.72 (1.30, 2.27)	0.034	48.7
**Research centre**				
Single centre	8	1.41 (1.09, 1.82)	0.009	26.3
Multicentre	4	1.61 (1.24, 2.11)	p<0.001	42.6
**Measurement method**				
biomarker	5	1.33 (1.07, 1.64)	0.233	26.9
Occupational assessment semi-definable scale	5	1.80 (1.34, 2.42)	0.061	46.3

OR, Odds Ratio; CI, Confidence Interval.

### Publication bias

**Fig. 4:**
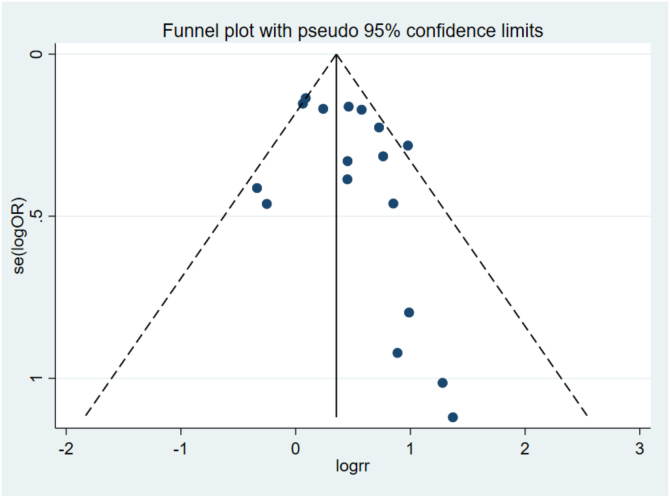
Funnel plot of the association between arsenic exposure and skin cancer risk.

Fig. 4 illustrates a funnel plot that shows the distribution of effect sizes from all studies, assessing publication bias. The plot displayed no notable asymmetry, as all studies were within the 95% Confidence Interval (95% CI) (Begg's p = 0.149). The results suggest that the analysis did not reveal any clear, significant bias in the publication.

## Discussion

This groundbreaking meta-analysis is the initial study to investigate the association between exposure to arsenic and the likelihood of developing skin cancer, encompassing both melanoma and non-melanoma varieties. Firstly, the research examined 12 papers, comprising prospective and case-control studies involving 48,003 participants, offering some evidence of an association between exposure to arsenic and a heightened likelihood of developing skin cancer. Secondly, the quantification of arsenic exposure in the reviewed literature presents distinct advantages, such as the precise identification of biomarkers, including arsenic concentrations in water, urine, and toenails. Additionally, the use of semi-quantitative three-level scales (low, medium, high) for quantification enhances the research potential of the data. Thirdly, previous studies that did not meet the authors’ criteria also supported these findings. For example, one study showed a significant association between inorganic Arsenic (iAs) and BCC, even at average water iAs concentrations below 40 ug/L.^45^ A separate investigation conducted in the United States revealed a 1.5 times increased likelihood of developing SCC due to exposure to arsenic in rice, in contrast to individuals who did not consume rice.^46^ Moreover, research carried out in Bangladesh through a cohort study established a direct association between the levels of arsenic in water and the occurrence of melanoma, BCC and SCC.^47^ In a meta-analysis, Shuai et al.^48^ found that arsenic exposure was associated with an increased risk of melanoma, with a pooled OR of 1.47 (95% CI 1.01–2.13). The results from previous studies, along with the meta-analysis, consistently indicate the possibility of a positive association between exposure to arsenic and the heightened risk of melanoma, BCC, and SCC. Additionally, the assessment using funnel plots and Begg's test did not show any significant evidence of small study effects. Subgroup and sensitivity analyses consistently indicated strong correlations.

The present study has several limitations. In the meta-analysis, the authors observed a notable degree of heterogeneity, which remained evident even following subgroup and sensitivity analyses. While such variability is not uncommon in similar studies, it is likely rooted in underlying differences across the literature, including population characteristics, adjustments for confounding variables, methodologies for determining outcomes, and approaches to assessing exposure. Due to the restricted quantity of research papers accessible, the authors were unable to perform stratified examinations according to levels of exposure to arsenic in the urine, arsenic in water, or exposure in the workplace. Additionally, combined estimates of arsenic concentrations across different exposure media may result in misclassification of exposure. Self-reported assessments through questionnaires are susceptible to memory bias, while data from biological samples, water, or air analysis may be subject to inaccuracies, potentially affecting the reliability of the association between arsenic exposure and the risk of developing skin cancer. Ultimately, personal protection measures, skin type, population mobility, and socioeconomic factors also represent significant potential confounding variables. The accuracy of a meta-analysis is contingent upon the quality of the underlying research. In case-control studies, key issues such as recall bias, varying measurement criteria, and residual confounders contribute to the risk of bias. While literature quality assessments and bias risk evaluations indicate that most studies have made adjustments for potential bias-inducing factors, the findings of meta-analyses may still be influenced. Due to the lack of high-quality studies that can effectively control for these bias factors, and because the results of studies with small sample sizes or statistical insignificance are often not easy to publish, the results of meta-analyses may still be affected and tend to report positive results. Therefore, the authors must be cautious in interpreting these results.

The precise molecular mechanisms involved in arsenic carcinogenesis are still being actively researched. It is widely acknowledged that arsenic exerts its toxicity through various pathways, such as inducing oxidative stress,^49^ immune dysfunction,^50^ genotoxicity,^51^ impairing DNA repair,^10^, ^52^ and disrupting signal transduction.^53^ It is believed that these procedures play a crucial role in the formation of skin cancer following arsenic exposure.

Arsenic triggers oxidative stress through the upregulation of Nicotinamide Adenine Dinucleotide Phosphate Oxidase (NADPHO), which results in the production of Reactive Oxygen Species (ROS). These ROS disturb the equilibrium of nitric oxide and glutathione, essential antioxidants, and affect other proteins responsible for redox homeostasis.^49^, ^54^ The formation of ROS triggers the activation of transcription factors like AP-1 and NF-κB, resulting in the excessive production of pro-inflammatory factors, which can enhance cell proliferation and potentially initiate carcinogenesis.^55^

Arsenic exposure activates the unfolded protein response signaling pathway, which impairs both innate and adaptive immunity by reducing immune cell function and number, creating a microenvironment conducive to tumor development.^51^ Within cells, arsenic metabolism involves key enzymes like Adenine nucleoside Methionine (SAM). Depletion of SAM due to arsenic-induced ROS leads to the methylation of inorganic arsenic into methylated forms. These methylated arsenic compounds further deplete SAM and can methylate DNA in an unregulated fashion, altering intracellular gene expression and potentially promoting carcinogenesis.^51^, ^56^ Additionally, arsenic disrupts cellular DNA repair mechanisms, such as base excision, mismatch repair and nucleotide excision, mainly by interfering with ATP and interacting with complexes likepoly-ADP ribose polymerase, O6-methyl-guanine-DNA methyltransferase and DNA polymerase β.^10^, ^52^ Impairment of these repair pathways may compromise genomic stability and disrupt normal cancer-related gene expression. Recent research indicates that arsenic triggers the Hippo pathway, which is critical for the survival and growth of cells. Arsenic modulates Hippo pathway activity by upregulating key proteins like Large Tumor Suppressor Kinase 1/2 (LATS1), Salvador homologue-1 (Sav1) and ste20-like kinase 1/2 (Mst1), which are known to play roles in various cancers, including skin cancer.^53^, ^57^

## Conclusions

This study demonstrates an association between arsenic exposure and the risk of skin cancer (both melanoma and non-melanoma), regardless of the exposure route ‒ whether through inhalation, ingestion, or dermal contact. This finding could have significant implications for patient education initiatives, enhancing public awareness of skin cancer prevention and consequently reducing both its health and economic burdens. Furthermore, it may serve as a valuable reference for professionals involved in mitigating occupational exposure. However, the study's limitations highlight the need for future research. There is a clear necessity for more rigorous prospective studies that can better control potential confounding variables to understand the precise impact of arsenic on skin cancer risk. Additionally, evaluating the response to different doses will provide a more robust scientific basis for creating regulations on arsenic levels in a variety of sources, including drinking water, soil, air, and food. Drawing from the latest studies, the authors’ recommendation is to initiate proactive measures aimed at not only validating arsenic as a contributing element to skin cancer but also crafting successful preventative tactics for areas plagued by chronic arsenic exposure.

## ORCID ID

Lijiao He: 0009-0004-7736-7342

Meiying Wei: 0009-0002-6913-8027

Qikui Yang: 0009-0009-1511-2199

Yun Huang: 0009-0005-1781-710X

Zuyuan Wei: 0009-0004-2411-3437

## Financial support

None declared.

## Authors' contributions

Lijiao He: Conceptualization; methodology; software; investigation; formal analysis; writing-original draft.

Meiying Wei: Data curation; software; validation.

Qikui Yang: Visualization.

Yun Huang: Software; validation.

Zuyuan Wei: Conceptualization; supervision; writing-review & editing.

## Conflicts of interest

None declared.

## References

[1] Boukamp P. (2005). Non-melanoma skin cancer: what drives tumor development and progression?. Carcinogenesis..

[2] Franceschi S., Levi F., Randimbison L., La Vecchia C. (1996). Site distribution of different types of skin cancer: new aetiological clues. Int J Cancer..

[3] Samarasinghe V., Madan V. (2012). Nonmelanoma skin cancer. J Cutan Aesthet Surg..

[4] Samarasinghe V., Madan V., Lear J.T. (2011). Management of high-risk squamous cell carcinoma of the skin. Expert Rev Anticancer Ther..

[5] Macbeth A.E., Grindlay D.J., Williams H.C. (2011). What’s new in skin cancer? An analysis of guidelines and systematic reviews published in 2008-2009. Clin Exp Dermatol..

[6] Zhang X.Y., Zhang P.Y. (2016). Genetics and epigenetics of melanoma. Oncol Lett..

[7] Nikolaou V., Stratigos A.J. (2014). Emerging trends in the epidemiology of melanoma. Br J Dermatol..

[8] Ho D., Lowenstein E.J. (2016). Fowler’s solution and the evolution of the use of arsenic in modern medicine. Skinmed..

[9] Piontek M., Hengels K.J., Borchard F., Strohmeyer G. (1989). [Noncirrhotic liver fibrosis afterchronic arsenic poisoning]. Dtsch Med Wochenschr..

[10] Yu H.S., Liao W.T., Chai C.Y. (2006). Arsenic carcinogenesis in the skin. J Biomed Sci..

[11] Issanov A., Adewusi B., Saint-Jacques N., Dummer T.J.B. (2024). Arsenic in drinking water and lung cancer: A systematic review of 35 years of evidence. Toxicol Appl Pharmacol..

[12] Sassano M., Seyyedsalehi M.S., Siea A.C., Boffetta P. (2023). Occupational arsenic exposure and genitourinary cancer: systematic review and meta-analysis. Occup Med (Lond)..

[13] Sassano M., Seyyedsalehi M.S., Siea A.C., Boffetta P. (2024). Occupational arsenic exposure and digestive and head and neck cancers: A systematic review and meta-analysis. Environ Res..

[14] Krewski D., Rice J.M., Bird M., Milton B., Collins B., Lajoie P. (2019). Concordance between sites of tumor development in humans and in experimental animals for 111 agents that are carcinogenic to humans. J Toxicol Environ Health B Crit Rev..

[15] IARC Working Group on the Evaluation of Carcinogenic Risks to Humans (2012). Arsenic, metals, fibres, and dusts. IARC Monogr Eval Carcinog Risks Hum..

[16] (2014). European Food Safety A. Dietary exposure to inorganic arsenic in the European population. EFSA Journal..

[17] Argos M., Kalra T., Pierce B.L., Chen Y., Parvez F., Islam T. (2011). A prospective study of arsenic exposure from drinking water and incidence of skin lesions in Bangladesh. Am J Epidemiol..

[18] Caldwell B.K., Smith W.T., Lokuge K., Ranmuthugala G., Dear K., Milton A.H. (2006). Access to drinking-water and arsenicosis in Bangladesh. J Health Popul Nutr..

[19] McCarty K.M., Hanh H.T., Kim K.W. (2011). Arsenic geochemistry and human health in South East Asia. Rev Environ Health..

[20] Molin M., Ulven S.M., Meltzer H.M., Alexander J. (2015). Arsenic in the human food chain, biotransformation and toxicology ‒ Review focusing on seafood arsenic. J Trace Elem Med Biol..

[21] Straif K., Benbrahim-Tallaa L., Baan R., Grosse Y., Secretan B., El Ghissassi F. (2009). A review of Human Carcinogens ‒ Part C: Metals, Arsenic, Dusts, and Fibres. Lancet Oncol..

[22] Kennedy C., Bajdik C.D., Willemze R., Bouwes Bavinck J.N. (2005). Chemical exposures other than arsenic are probably not important risk factors for squamous cell carcinoma, basal cell carcinoma and malignant melanoma of the skin. Br J Dermatol..

[23] Surdu S., Fitzgerald E.F., Bloom M.S., Boscoe F.P., Carpenter D.O., Haase R.F. (2013). Occupational exposure to arsenic and risk of nonmelanoma skin cancer in a multinational European study. Int J Cancer..

[24] Mitropoulos P., Norman R. (2005). Occupational nonsolar risk factors of squamous cell carcinoma of the skin: a population-based case-controlled study. Dermatol Online J.

[25] Sánchez G., Nova J. (2013). Risk factors for squamous cell carcinoma, a study by the National Dermatology Centre of Colombia. Actas Dermosifiliogr..

[26] Suárez B., López-Abente G., Martínez C., Navarro C., Tormo M.J., Rosso S. (2007). Occupation and skin cancer: the results of the HELIOS-I multicenter case-control study. BMC public health.

[27] Karagas M.R., Stukel T.A., Morris J.S., Tosteson T.D., Weiss J.E., Spencer S.K. (2001). Skin cancer risk in relation to toenail arsenic concentrations in a US population-based case-control study. Am J Epidemiol..

[28] Gilbert-Diamond D., Li Z., Perry A.E., Spencer S.K., Gandolfi A.J., Karagas M.R. (2013). A population-based case-control study of urinary arsenic species and squamous cell carcinoma in New Hampshire. USA. Environ Health Perspect..

[29] Smith A.H., Goycolea M., Haque R., Biggs M.L. (1998). Marked increase in bladder and lung cancer mortality in a region of Northern Chile due to arsenic in drinking water. Am J Epidemiol..

[30] Cebrián M.E., Albores A., Aguilar M., Blakely E. (1983). Chronic arsenic poisoning in the north of Mexico. Hum Toxicol..

[31] Tseng W.P., Chu H.M., How S.W., Fong J.M., Lin C.S., Yeh S. (1968). Prevalence of skin cancer in an endemic area of chronic arsenicism in Taiwan. J Natl Cancer Inst..

[32] Hsueh Y.M., Chiou H.Y., Huang Y.L., Wu W.L., Huang C.C., Yang M.H. (1997). Serum beta-carotene level, arsenic methylation capability, and incidence of skin cancer. Cancer Epidemiol Biomarkers Prev..

[33] Bedaiwi A., Wysong A., Rogan E.G., Clarey D., Arcari C.M. (2022). Arsenic Exposure and Melanoma Among US Adults Aged 20 or Older. 2003-2016. Public Health Rep..

[34] Langston M.E., Brown H.E., Lynch C.F., Roe D.J., Dennis L.K. (2022). Ambient UVR and Environmental Arsenic Exposure in Relation to Cutaneous Melanoma in Iowa. Int J Environ Res Public Health..

[35] Beane Freeman L.E., Dennis L.K., Lynch C.F., Thorne P.S., Just C.L. (2004). Toenail arsenic content and cutaneous melanoma in Iowa. Am J Epidemiol..

[36] Collatuzzo G., Boffetta P., Dika E., Visci G., Zunarelli C., Mastroeni S. (2023). Occupational exposure to arsenic, mercury and UV radiation and risk of melanoma: a case-control study from Italy. Int Arch Occup Environ Health..

[37] Dennis L.K., Lynch C.F., Sandler D.P., Alavanja M.C. (2010). Pesticide use and cutaneous melanoma in pesticide applicators in the agricultural heath study. Environ Health Perspect..

[38] Yager J.W., Erdei E., Myers O., Siegel M., Berwick M. (2016). Arsenic and ultraviolet radiation exposure: melanoma in a New Mexico non-Hispanic white population. Environ Geochem Health..

[39] Page M.J., McKenzie J.E., Bossuyt P.M., Boutron I., Hoffmann T.C., Mulrow C.D. (2021). The PRISMA 2020 statement: an updated guideline for reporting systematic reviews. Syst Rev.

[40] Stang A. (2010). Critical evaluation of the Newcastle-Ottawa scale for the assessment of the quality of nonrandomized studies in meta-analyses. Eur J Epidemiol..

[41] Sterne J.A., Hernán M.A., Reeves B.C., Savović J., Berkman N.D., Viswanathan M. (2016). ROBINS-I: a tool for assessing risk of bias in non-randomised studies of interventions. BMJ..

[42] Borenstein M., Hedges L.V., Higgins J.P., Rothstein H.R. (2010). A basic introduction to fixed-effect and random-effects models for meta-analysis. Res Synth Methods..

[43] Higgins J.P., Thompson S.G. (2002). Quantifying heterogeneity in a meta-analysis. Stat Med..

[44] Begg C.B., Mazumdar M. (1994). Operating characteristics of a rank correlation test for publication bias. Biometrics..

[45] Leonardi G., Vahter M., Clemens F., Goessler W., Gurzau E., Hemminki K. (2012). Inorganic arsenic and basal cell carcinoma in areas of Hungary, Romania, and Slovakia: a case-control study. Environ Health Perspect..

[46] Gossai A., Zens M.S., Punshon T., Jackson B.P., Perry A.E., Karagas M.R. (2017). Rice Consumption and Squamous Cell Carcinoma of the Skin in a United States Population. Environ Health Perspect..

[47] Choudhury M.I.M., Shabnam N., Ahsan T., Ahsan S.M.A., Kabir M.S., Khan R.M. (2018). Cutaneous Malignancy due to Arsenicosis in Bangladesh: 12-Year Study in Tertiary Level Hospital. Biomed Res Int.

[48] Shuai W., Huang Q., Xu L., Mu Y. (2024). Association between arsenic exposure and melanoma: a meta-analysis. Int J Dermatol..

[49] Kumagai Y., Sumi D. (2007). Arsenic: signal transduction, transcription factor, and biotransformation involved in cellular response and toxicity. Annu Rev Pharmacol Toxicol..

[50] Grivennikov S.I., Greten F.R., Karin M. (2010). Immunity, inflammation, and cancer. Cell..

[51] Mahata J., Basu A., Ghoshal S., Sarkar J.N., Roy A.K., Poddar G. (2003). Chromosomal aberrations and sister chromatid exchanges in individuals exposed to arsenic through drinking water in West Bengal. India. Mutat Res..

[52] Vahidnia A., van der Voet G.B., de Wolff F.A. (2007). Arsenic neurotoxicity--a review. Hum Exp Toxicol..

[53] Zhao B., Ye X., Yu J., Li L., Li W., Li S. (2008). TEAD mediates YAP-dependent gene induction and growth control. Genes Dev..

[54] Kumagai Y. (2009). [Fusion of field and laboratory studies on the investigation of arsenic]. Yakugaku Zasshi..

[55] Kitchin K.T., Ahmad S. (2003). Oxidative stress as a possible mode of action for arsenic carcinogenesis. Toxicol Lett..

[56] Niedzwiecki M.M., Hall M.N., Liu X., Oka J., Harper K.N., Slavkovich V. (2013). A dose-response study of arsenic exposure and global methylation of peripheral blood mononuclear cell DNA in Bangladeshi adults. Environ Health Perspect..

[57] Halder G., Johnson R.L. (2011). Hippo signaling: growth control and beyond. Development.

